# Massive Pyometra Due to Senile Endometritis in a Postmenopausal Woman: A Rare Entity

**DOI:** 10.7759/cureus.32775

**Published:** 2022-12-21

**Authors:** Tejal Waghe, Neema Acharya, Jyotsana Potdar, Shazia Mohammad

**Affiliations:** 1 Department of Obstetrics and Gynaecology, Jawaharlal Nehru Medical College, Datta Meghe Institute of Medical Science (Deemed to be University), Wardha, IND

**Keywords:** post-menopause, rupture uterus, endometritis, senile, draining pus, pyometra

## Abstract

An 83-year-old postmenopausal female P5L5 (all full-term normal deliveries) presented with complaints of foul-smelling purulent discharge per vagina for 15 days associated with pain in abdomen. A midline mass was palpable per abdomen in the suprapubic region corresponding to 16 weeks size gravid uterus, which was soft to firm in consistency. On examination per vaginum, the atrophied cervix was found flush with the vagina and purulent discharge was seen draining through the cervix. Blood reports showed raised total leucocyte count with granulocyte predominance. Abdominal ultrasonography revealed a uterine cavity filled with echogenic contents, with no abdominal cavity collection. The patient was started on IV antibiotics and planned for dilatation and curettage. On histopathology acute on chronic senile endometritis was found with no evidence of malignant cells. Tuberculosis gene testing was found to be negative. We conclude that the senile endometritis leading to cervical stenosis as seen during dilatation and curettage had led to the pyometra and no evidence of malignancy was found.

## Introduction

According to a study by Lee DH et al., pyometra, which is a deposit of pus in the uterine cavity, has an incidence of 0.01-0.5% in gynecologic patients [1]. In older patients, the incidence rises with age and is 13.6%. A study by Chauhan A et al. shows the median age of presentation to be 65 years, and only less than one-third of cases are linked to an underlying malignancy [2]. Some other causes of such huge pyometra may be foreign bodies, puerperal infections, and uterine anomalies [3]. Bacteremia, sepsis, and spontaneous uterine rupture that results in widespread peritonitis are the three main adverse effects of pyometra. The preferred course of treatment is cervical dilatation and pus drainage with appropriate antibiotic protection [1]. Pyometra does not respond well to the standard selection of antibiotics. Postmenopausal cervical stenosis can make pyometra draining challenging, as seen in a study by Singhal et al. [4]. Here, we report the case of a patient in whom, due to senile endometritis with cervical stenosis, gross pyometra was formed of non-tubercular origin and presented with pus discharge per vaginum.

## Case presentation

An 83-year-old female was admitted to our hospital with the chief complaints of purulent discharge per vaginum for 15 days, which was associated with lower abdominal pain. The discharge was foul smelling, yellow in color, mucopurulent, and copious in amount. It was not associated with itching and the pain was insidious in onset and dull aching and localized to the lower abdomen and aggravated on work while relieved on medications. There was no history of similar complaints in the past. There was no history of vaginal bleeding.

**Figure 1:**
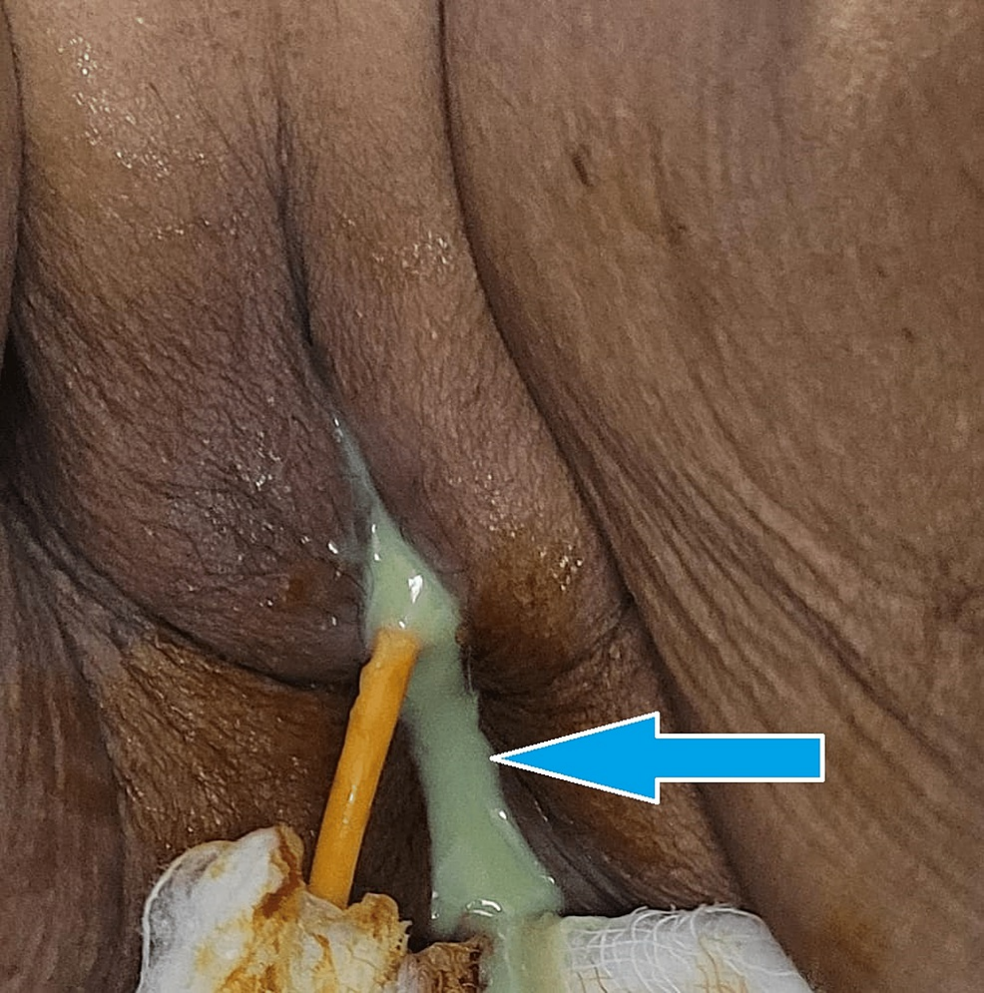
Frank pus discharge from vaginal opening.

The patient was a known case of hypertension for the past 10 years and was on regular medication. There was no history of diabetes, tuberculosis, or other major medical or surgical illness in past. She had undergone tubal ligation 40 years back. She was menopausal for 33 years and had five full-term normal deliveries. Bowel and bladder habits were normal and normal sleep and appetite. On examination she was afebrile, her pulse was 82 beats per minute, regular in rate and rhythm, respiratory rate was 18 breaths per minute, and blood pressure was 122/80 mm of Hg. The central nervous system, cardiovascular system, and respiratory system examination findings were found to be normal. A midline mass was palpable per abdomen in the suprapubic region corresponding to 16 weeks size gravid uterus The mass was tender soft to firm in consistency having restricted mobility. On per speculum examination, both labia were fused partially in the posterior end (postmenopausal adhesion), which could be easily separated with blunt finger dissection done gently. Per speculum examination revealed the vagina full of foul-smelling purulent discharge, which was copious in amount (Figure 1). A vaginal examination revealed an atrophied, hard cervix that was flush with the vagina. The abdominal mass was felt continuous with the cervix and cervical movements were transmitted to the mass suggestive of uterine origin. Both fornices were free of any palpable pathology. A clinical diagnosis of pyometra was made.

At the time of admission, blood tests showed a hemoglobin level of 10.2 g%, a total leukocyte count of 11,400/mm^3^, a differential leukocyte count of 75% polymorphs, 20% lymphocytes, 3% monocytes, and 2% eosinophils, as well as levels of 22 mg/dL of urea and 0.8 mg/dL of creatinine. Both the chest X-ray and the ECG were clear.

**Figure 2:**
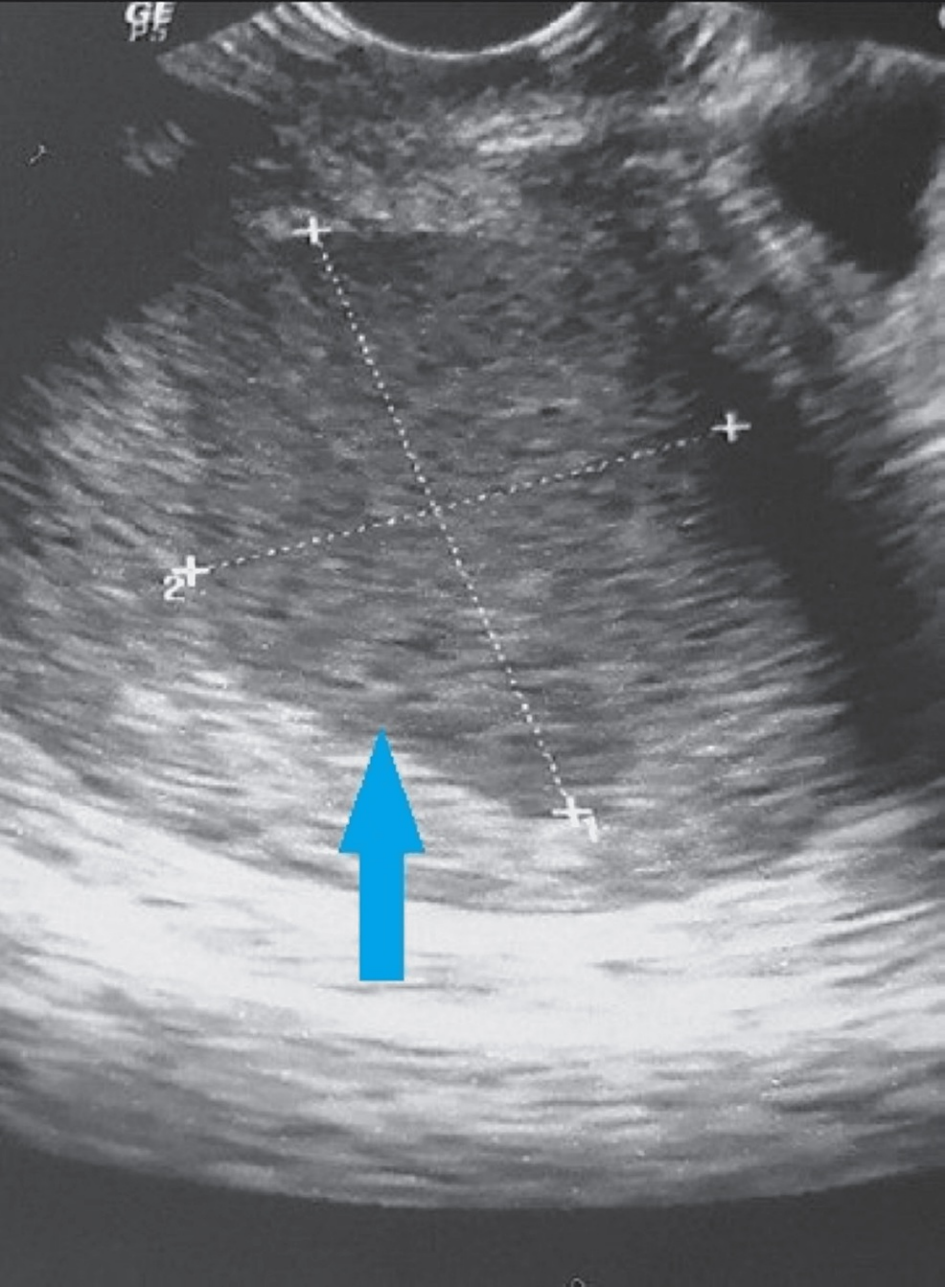
Transabdominal USG image of the uterus show uterine cavity filled with echogenic content (arrow).

Ultrasonography of the abdomen and pelvis revealed an enlarged uterus with fluid collection corresponding to an echogenic shadow in the cavity suggestive of hydrometra/hematometra/pyometra (Figure 2). Vaginal pus culture showed 1-2/hpf epithelial cells seen, pus cells present, and no trophozoite was seen.

Pus culture was suggestive of *Escherichia coli* growth, sensitive to ceftriaxone, amoxycillin, amikacin, piperacillin, and gentamycin. The patient was started on injectable antibiotic ceftriaxone 1 gm 12 hourly and injection of metronidazole 100 mg eight hourly for 72 hours till dilatation and curettage were done after 72 hours, which showed a closed cervix. Post procedure, clinically the uterine mass reduced and ultrasound showed an empty uterine cavity. Due to active infection, hysteroscopy could not be done; hence, dilatation and curettage were done and an endometrial sample was sent for histopathological examination. Endometrial histopathology revealed infiltration of lymphocytes, plasma cells, extensive hemorrhage, and scanty endometrial glands admixed with few polymorphs, which favors the diagnosis of acute on chronic endometritis on histology. There was no evidence of malignant cells. Endometrial samples sent for polymerase chain reaction for *Mycobacterium tuberculosis* (TB-PCR) came out negative.

## Discussion

Pyometra is a condition in which the uterine cavity is filled with purulent content. Most of the time it is because of cervical stenosis due to cervical malignancy, but in this case, the senile endometritis associated with cervical stenosis led to pyometra. The common causes of pyometra in postmenopausal age include malignancy of the genital system including post-radiotherapy complications, benign conditions such as an endometrial polyp, senile endometritis, idiopathic conditions leading to cervical stenosis, or postoperative infection. Out of these, 22.2% of cases are associated with malignancies, 3.7% with genital tract abnormalities, and 74.1% are due to idiopathic causes [3]. The classic triad of clinical manifestations includes lower stomach pain, postmenopausal hemorrhage per vaginum, and purulent vaginal discharge. If nothing is suspected and specifically looked for, the diagnosis is difficult. In order to rule out related cancers, a thorough history and clinical examination should be performed after the diagnosis has been made. It is a significant medical disorder with potential life-threatening implications like spontaneous uterine perforation [2]. Prognosis in cases of perforated pyometra is variable. Those cases not associated with malignancy have a better prognosis compared to those that are associated with malignancy as seen in previous studies [5,6]. Proper antibiotic coverage should be given, and the procedure of choice remains dilatation and curettage, as also concluded in the review by Chan et al. [7]. 

Furthermore, the endometrial samples should undergo a histopathological examination to rule out malignancy in the form of cervical cancer [8,9]. The pus collected should be cultured and sensitivity testing to be done for specific antibiotic coverage and tubercular gene testing should also be done. Hysterectomy may sometimes be indicated, as in perforation and sepsis. Recurrence is common in 2-11 months; therefore, in pyometra due to senile endometritis, after adequate drainage, pan hysterectomy should be done within one month, when fit for surgery. For those patients who are not fit enough for surgery, medical management using cyclic estrogen therapy (Premarin 0.625 mg daily) for four to six months is beneficial [10].

## Conclusions

Pyometra is a serious medical condition, which should be diagnosed and treated as early as possible. Usually in a massive pyometra at this age, one may consider malignancy as the only cause, but benign conditions such as senile endometritis should be kept in mind while working towards a diagnosis. Malignancy should be ruled out in every case and care must be taken to prevent and manage uterine perforation. USG is the first and most important investigation to quantify and tell the type of fluid collected in the uterus and rule out uterine perforation. CT scan and MRI should also be considered for quantification of collection and to rule out pyoperitoneum.
